# Effect and process evaluation of a kindergarten-based, family-involved cluster randomised controlled trial in six European countries on four- to six-year-old children’s steps per day: the ToyBox-study

**DOI:** 10.1186/s12966-017-0574-z

**Published:** 2017-08-29

**Authors:** Marieke De Craemer, Maïté Verloigne, Ilse De Bourdeaudhuij, Odysseas Androutsos, Violeta Iotova, Luis Moreno, Berthold Koletzko, Piotr Socha, Yannis Manios, Greet Cardon, on behalf of the ToyBox-study group, Yannis Manios, Berthold Koletzko, Ilse De Bourdeaudhuij, Mai Chin A. Paw, Luis Moreno, Carolyn Summerbell, Tim Lobstein, Lieven Annemans, Goof Buijs, John Reilly, Boyd Swinburn, Dianne Ward, Odysseas Androutsos, Eva Grammatikaki, Christina Katsarou, Eftychia Apostolidou, Eirini Efstathopoulou, Kristin Duvinage, Sabine Ibrügger, Angelika Strauß, Birgit Herbert, Julia Birnbaum, Annette Payr, Christine Geyer, Greet Cardon, Marieke De Craemer, Ellen De Decker, Stefaan De Henauw, Lea Maes, Carine Vereecken, Jo Van Assche, Lore Pil, Saskia te Velde, Theodora Mouratidou, Juan Fernandez, Maribel Mesana, Pilar De Miguel-Etayo, Esther González, Luis Gracia-Marco, Beatriz Oves, Agneta Yngve, Susanna Kugelberg, Christel Lynch, Annhild Mosdøl, Helen Moore, Wayne Douthwaite, Catherine Nixon, Susanne Kreichauf, Andreas Wildgruber, Piotr Socha, Zbigniew Kulaga, Kamila Zych, Magdalena Góźdź, Beata Gurzkowska, Katarzyna Szott, Violeta Iotova, Mina Lateva, Natalya Usheva, Sonya Galcheva, Vanya Marinova, Zhaneta Radkova, Nevyana Feschieva, Annemiek Dorgelo, Aviva Nethe, Jan Jansen, Otto Gmeiner, Jutta Retterath, Julia Wildeis, Axel Günthersberger, Leigh Gibson, Claus Voegele

**Affiliations:** 1 Department of Movement and Sport Sciences, Ghent University, Watersportlaan 2, 9000 Ghent, Belgium; 2 Research Foundation Flanders, Brussels, Belgium; 3 Department of Nutrition and Dietetics, Harokopio University, School of Health Science & Education, E. Venizelou 70, 17671 Athens, Greece; 4 Medical University Varna, Clinic of Paediatric Endocrinology, UMHAT “St. Marina”, “Hr. Smirnenski” Blvd, Varna, Bulgaria; 5 University of Zaragoza, GENUD (Growth, Exercise, Drinking behaviour and Development), C/Corona de Aragón 42, 50009 Zaragoza, Spain; 6 University of Munich Medical Centre, Dr. von Hauner Children’s Hospital, Lindwurmstr.4, 80337 Munich, Germany; 7 Children’s Memorial Institute, Al. Dzieci Polskich 20, 04–730 Warsaw, Poland

**Keywords:** Effect evaluation, Process evaluation, Preschool, Europe, Pedometer, Step counts, ToyBox, RCT

## Abstract

**Background:**

The ToyBox-intervention is a theory- and evidence-based intervention delivered in kindergartens to improve four- to six-year-old children’s energy balance-related behaviours and prevent obesity. The current study aimed to (1) examine the effect of the ToyBox-intervention on increasing European four- to six-year-old children’ steps per day, and (2) examine if a higher process evaluation score from teachers and parents was related to a more favourable effect on steps per day.

**Methods:**

A sample of 2438 four- to six-year-old children (51.9% boys, mean age 4.75 ± 0.43 years) from 6 European countries (Belgium, Bulgaria, Germany, Greece, Poland and Spain) wore a motion sensor (pedometer or accelerometer) for a minimum of two weekdays and one weekend day both at baseline and follow-up to objectively measure their steps per day. Kindergarten teachers implemented the physical activity component of the ToyBox-intervention for 6 weeks in total, with a focus on (1) environmental changes in the classroom, (2) the child performing the actual behaviour and (3) classroom activities. Children’s parents received newsletters, tip cards and posters. To assess intervention effects, multilevel repeated measures analyses were conducted for the total sample and the six intervention countries separately. In addition, process evaluation questionnaires were used to calculate a total process evaluation score (with implementation and satisfaction as a part of the overall score) for teachers and parents which was then linked with the physical activity outcomes.

**Results:**

No significant intervention effects on four- to six-year-old children’ steps per weekday, steps per weekend day and steps per average day were found, both in the total sample and in the country-specific samples (all *p* > 0.05). In general, the intervention effects on steps per day were least favourable in four- to six-year-old children with a low teachers process evaluation score and most favourable in four- to six-year-old children with a high teachers process evaluation score. No differences in intervention effects were found for a low, medium or high parents’ process evaluation score.

**Conclusion:**

The physical activity component of the ToyBox-intervention had no overall effect on four- to six-year-old children’ steps per day. However, the process evaluation scores showed that kindergarten teachers that implemented the physical activity component of the ToyBox-intervention as planned and were satisfied with the physical activity component led to favourable effects on children’s steps per day. Strategies to motivate, actively involve and engage the kindergarten teachers and parents/caregivers are needed to induce larger effects.

**Electronic supplementary material:**

The online version of this article (10.1186/s12966-017-0574-z) contains supplementary material, which is available to authorized users.

## Background

Sufficient levels of physical activity (PA) are associated with numerous positive mental and physical health outcomes, even at preschool age [1]. In addition, PA plays an important role in the prevention of overweight and obesity, and PA tracks from year to year [2–4]. However, many preschool children appear to be insufficiently active [5–9] and do not comply with the PA guidelines of 180 min of total PA per day [10, 11]. For this reason, several interventions targeting an increase in preschoolers’ PA have been developed and implemented, with mixed effects as a result [12–18].

The aim of the ToyBox-study was to develop, implement and evaluate a kindergarten-based, family-involved intervention to prevent overweight and obesity in four- to six-year-old preschool children from six European countries (i.e., Belgium, Bulgaria, Germany, Greece, Poland, and Spain). One of the four behaviours on which the intervention focused, was PA (together with sedentary behaviour, water consumption, and snacking behaviour) [19, 20]. The effectiveness of the intervention on objectively measured PA (via accelerometers) has already been evaluated in the Belgian sample, as accelerometers were only used in Belgian preschool children. More specifically, small but positive effects were found in sub-groups such as preschool boys and preschoolers from kindergartens with a high socio-economic status (SES) [21]. However, the effectiveness of the ToyBox-intervention on steps per day in the total sample still has to be investigated. Further, as preliminary results showed that there was a big variance in implementation of the different ToyBox components across kindergartens [22, 23], the effectiveness will be studied as a function of the variability of the implementation of the PA-component of the intervention. Therefore, it is important to examine the relationship between effect evaluation (study outcomes) and process evaluation (intervention implementation and participant satisfaction) in order to better understand the potential effects of a health promotion intervention [24].

A process evaluation should be guided by using a specific framework. The model used in the ToyBox-intervention is the model by Saunders et al. (2005) and is described in detail elsewhere [25, 26]. This model describes and recommends several key elements to conduct a process evaluation, such as *reach* (level of participation in the intervention), *fidelity* (quality of implementation), *dose delivered* (the amount of the intervention that was delivered by the implementers), *dose received – exposure* (the level of active participation and being receptive to or using the materials and resources), *dose received – satisfaction* (the level of satisfaction of the implementer and the target group regarding the intervention) and *context* (within which the ToyBox-intervention was implemented) [25].

Thus, the aim of the current study was (1) to evaluate the effectiveness of the ToyBox-intervention on European preschoolers’ objectively measured steps per day in the total sample and in the country-specific samples, and (2) to examine whether a higher level of intervention implementation and satisfaction of the PA-component of the ToyBox-intervention was related to more favourable effects on preschoolers’ steps per day in the total sample.

## Methods

### Study protocol

The kindergarten-based ToyBox-intervention with family involvement, targeting four- to six-year-old preschoolers, has a cluster randomised pre-test post-test design with intervention kindergartens and control kindergartens across six European countries: Belgium, Bulgaria, Germany, Greece, Poland, and Spain. Preschool children and their parents/caregivers were recruited at kindergartens, daycare centers or preschool settings, depending on the country regulations and legislation. In Germany, Bulgaria, Spain and Poland, children/families were recruited from kindergartens, in Greece from kindergartens and daycare centers, and in Belgium from preschool settings. In order to avoid confusion for the reader, all these settings (kindergartens, daycare centers, preschool settings) will be referred to as “kindergartens” in this paper. Additionally, to avoid confusion regarding the term “preschool children” we will use the term “four- to six-year-old children” throughout the manuscript.

**Fig. 1:**
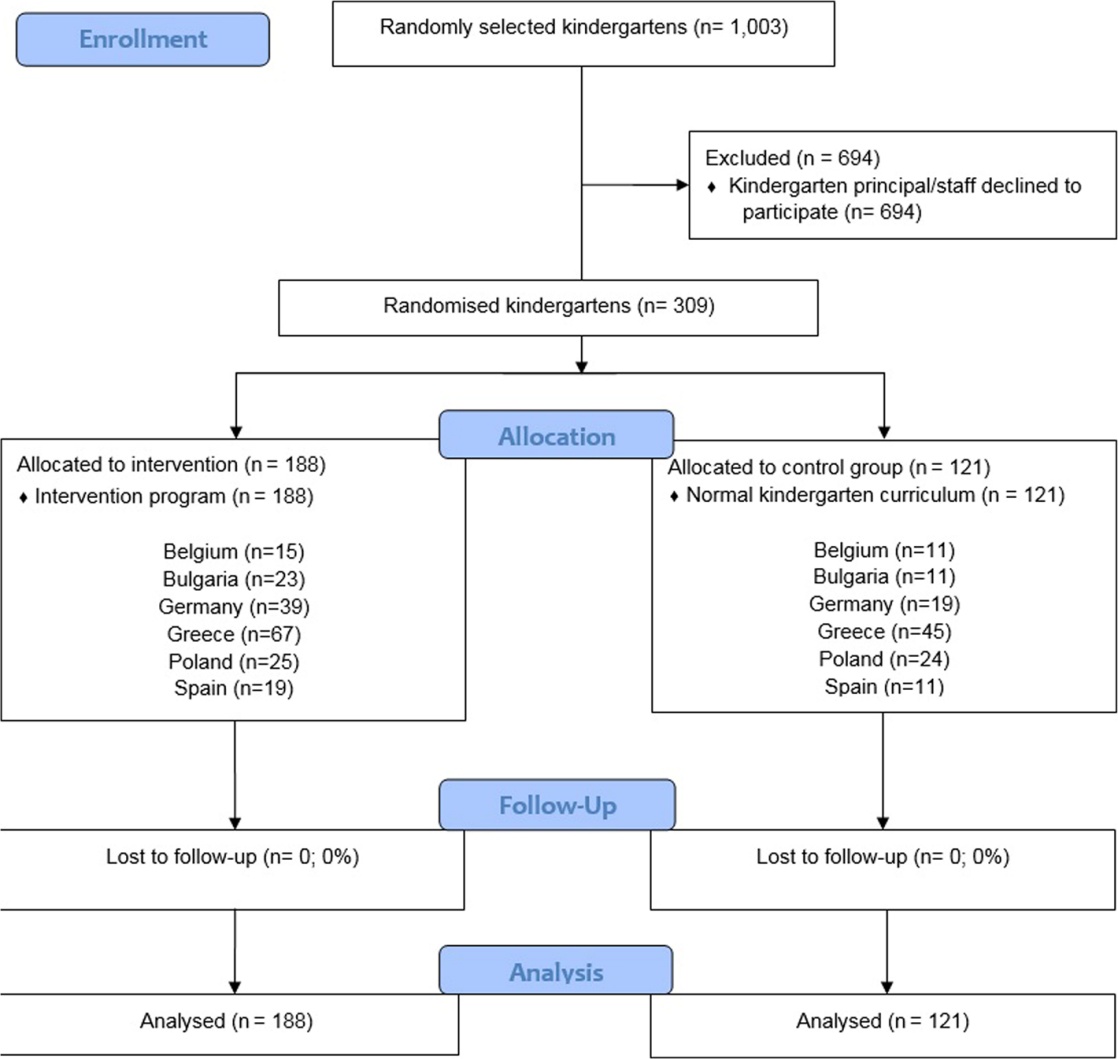
Flow chart of included kindergartens into the intervention

**Fig. 2:**
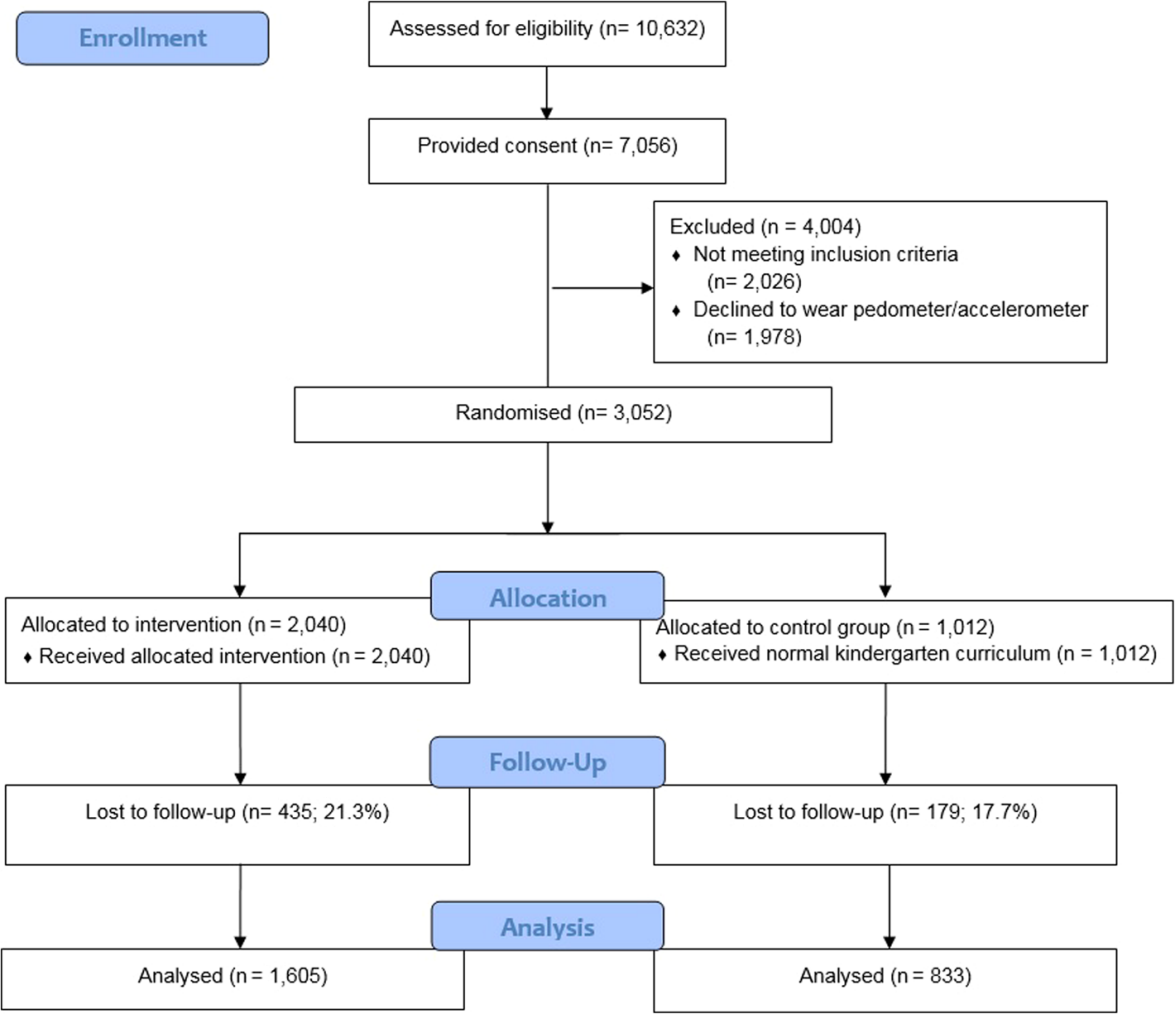
Flow chart of included four- to six-year-old children with valid pedometer data into the intervention

Kindergartens were selected in the provinces of West- and Oost-Vlaanderen in Belgium, Varna in Bulgaria, Bavaria in Germany, Attica in Greece, Mazowiecki in Poland, and Zaragoza in Spain. Kindergartens were recruited from different SES backgrounds. Lists of all municipalities that exist within the selected provinces were collected and information on the SES variables was provided (mean years of education for the population aged 25–55 years or annual income). Tertiles were created, based on the selected SES variables, and five municipalities were randomly selected from each tertile (i.e., approximately five municipalities for low SES, five for medium SES, and five for high SES) in each country. Then, 1003 kindergartens within these randomly chosen municipalities across the six countries were randomly selected (with the exclusion of the lowest 20% of the kindergartens with the smallest number of children), and a personal visit was performed to inform the kindergarten staff about the ToyBox-study. In total, 309 kindergartens (30.8%) across the six countries agreed to participate in the study, and all four- to six-year-old children born in 2007 and 2008 (*n* = 16,798; age at baseline was 3.5–5.5 years old) received an information letter to take home in which the purpose of the study was explained to the parents/caregivers, and the child was invited to wear a pedometer or accelerometer for six consecutive days. Sample size calculations can be found elsewhere [27]. The flow of kindergartens through the study is depicted in Fig. 1, and the flow of participants through the study is depicted in Fig. 2. In total, 7056 parents/caregivers (42.0%) provided consent for their child to participate in the study.

After the recruitment of the kindergartens and the execution of the baseline measurements, kindergartens’ municipalities were randomly assigned to the intervention or control condition (ratio 2:1) to avoid contamination between kindergartens in the same municipality. This was done by by the project coordinator (Greece) with the use of a command in Excel, which means that the randomisation occurred automatically and electronically. Kindergartens allocated to the intervention condition received the intervention programme. Kindergartens allocated to the control condition continued with the normal kindergarten curriculum. The ToyBox-intervention was implemented within the school year 2012–2013.

Before the start of the intervention, baseline measurements were performed on weekdays from May to June 2012. On those days, researchers visited intervention and control kindergartens and fitted those preschool children with an accelerometer (Belgium) or a pedometer (other countries) for whom written informed consent from their parents/caregivers had been obtained. One year later, from May to June 2013, follow-up measurements were performed and again, the same children with written informed consent from both intervention and control kindergartens received a pedometer or accelerometer to objectively measure their step counts.

### Ethical statement

The ToyBox-study was approved by the Ethical Committees in all European countries, in line with national regulations (i.e. in Belgium by the Medical Ethics Committee of the Ghent University Hospital; in Bulgaria by the Ethics Committee of the Medical University of Varna; in Germany by the Ethics Committee of the Ludwig Maximilian University of Munich; in Greece by the Bioethics Committee of Harokopio University and the Greek Ministry of Education; in Poland by the Bioethics Committee of the Children’s Memorial Health Institute and the Department of Information and Publicity of the Polish Ministry of Education; and in Spain by the Clinical Research Ethics Committee and the Department of Consumers’ Health of the Government of Aragón. The ToyBox-study is registered with the clinical trials registry clinicaltrials.gov, ID: NCT02116296.

### ToyBox-intervention: PA-component

The kindergarten-based, family-involved ToyBox-intervention was planned and developed following the Intervention Mapping protocol [28, 29]. The intervention included components on water consumption, healthy snacking and sedentary behaviour, as well as a PA-component which will be further discussed below.

The ToyBox-intervention consisted of 24 intervention weeks. The PA-part of the intervention was implemented in weeks 5 until 8 (i.e., ‘first focus’), and had a two-week ‘repetition period’ in weeks 19 and 20. Furthermore, some PA-components were also implemented throughout the whole school year, such as restructuring the classroom (e.g., rearranging the furniture to create space to be physically active, providing equipment (not provided by the researchers) in the classroom that stimulates moving such as balls, big sponge bricks,…) and installing movement landscapes (e.g. large equipment such as wall bars, benches, mats, hoops,…) in the classroom. The ToyBox-intervention was implemented by the kindergarten teachers, who had two 1-h teacher training sessions with the researchers to explain the goals and the material of the ToyBox-study, and to answer kindergarten teachers’ questions, prior to the intervention. During the second training session, teachers were provided with the “ToyBox”, i.e. a box containing material for the class (including a teachers’ guide, four classroom activity guides and a kangaroo hand puppet), and material for the home (including newsletters, tip-cards, and posters for the parents/caregivers). In the classroom activity guide for PA, three different themes were included, namely (1) setting environmental changes in the classroom (i.e., how to rearrange the classroom to make free space for the preschool children to be more active), (2) the preschool child performing the actual behaviour (i.e., being physically active during structured physical education sessions), and (3) classroom activities (i.e., kangaroo stories, and PA excursions). Teachers were asked to allocate a minimum of 1 h per week to use the ToyBox-materials and to implement the ToyBox-intervention in the classroom, while the environmental changes were conducted and retained throughout the project. The 1 h per week recommendation was chosen based on the focus groups that were held before the development of the intervention [30]. Kindergarten teachers clearly mentioned that they already have a busy week schedule and that they only wanted to implement an intervention when it would include ready-to-use materials and when they only had to devote limited time to the implementation. For that reason, we only included 60 min per week as a minimum recommendation, although devoting more time per week or even per day was recommended and encouraged.

To involve the parents/caregivers, four- to six-year-old children received two newsletters, two tip-cards and one poster (with key messages on PA that could be coloured at kindergarten or at home) to take home for their parents/caregivers. The newsletters and tip-cards contained tips and strategies to increase four- to six-year-old children’ PA levels. The relevant materials can be found on the ToyBox-website (www.toybox-study.eu).

### Instrumentation

PA was assessed by means of steps per day using Omron Walking Style Pro pedometers (HJ-720IT-E2) (Bulgaria, Germany, Greece, Poland, and Spain) and ActiGraph (Pensacola, FL) accelerometers with activated step count function (Belgium). Step counts from the pedometer and accelerometer are comparable and have been validated to measure PA in preschool children [31]. The devices were worn on the right hip, secured by an elastic waist band. Pedometers were only used as a measuring instrument during the baseline and follow-up measurements and not as a monitoring instrument.

### Procedure

Four- to six-year-old children wore a motion sensor (pedometer or accelerometer) for six full consecutive days, including two weekend days. Accelerometers were initialised to measure activity counts and step counts in 15-s epochs. Four- to six-year-old children’s parents/caregivers received an information letter and were instructed to let their child wear the motion sensor during all waking hours and to remove it only during water-based activities. After data collection, data from pedometers were downloaded using Omron Health Management Software version E1.012, and data from accelerometers were downloaded using ActiLife version 5.5.5-software. Both the first (fitting day) and sixth day (collection day) were omitted, because these days did not have a full day of data and were therefore incomplete. All step counts below 1000 and above 30,000 steps per day were deleted and treated as missing data, which is according to the rules of Rowe et al. (2014) [32]. Four- to six-year-old children’s step count data were included in data analyses when they had valid data for a minimum of two weekdays and one weekend day.

### Process evaluation: teachers

To investigate the intervention process and satisfaction, teachers had to fill in two logbooks with specific questions regarding the process and satisfaction of the PA component, which were based on the model of Saunders et al. (2005) [25, 26]. The key process evaluation elements that were questioned in the monthly logbooks were “Fidelity”, “Dose delivered”, and “Dose received – satisfaction” [25, 26]. No information on “Reach”, “Dose received – exposure” and “Context” was available.

The specific questions per key element that were used from the monthly logbooks are depicted in Table 1 with an explanation about the score. Teachers filled in a logbook during the “first focus” and during the “repetition period” [33]. This logbook was completed either via phone calls from the researchers to the teachers or via email by the teachers. The questions with answer possibilities on a 5-point-scale were dichotomised based on the mean score. Below the mean was coded into 0, and equal to or higher than the mean was coded into 1. All monthly logbook questions with a score of 0 or 1 were used to calculate the total process evaluation score for each intervention kindergarten by adding up all scores. A higher process evaluation score represents a higher level of intervention implementation and satisfaction. The minimum score was 0 and the maximum score was 26. Based on the teachers’ process evaluation score, kindergartens were divided into three groups, based on the tertiles: kindergartens with a low (recoded into 1), a medium (recoded into 2) or a high level (recoded into 3) of implementation and satisfaction. To look for a difference in effect between the intervention group (with three categories of teachers’ process evaluation scores: 1, 2, 3) and the control group, the control group (recoded into 0) was added as an extra group (in addition to low, medium and high process evaluation scores).

**Table 1:** Overview process evaluation questions to calculate the process evaluation score (score on 26) for teachers

	Fidelity = whether the intervention was delivered as intended by the teachers and received by the children	Dose delivered = how much was delivered by the teachers and received by the children	Dose received – satisfaction = how the intervention was received by the teachers
Questionnaire Teachers’ physical activity logbook (first focus)	*“When did you deliver the 1st physical activity behaviour newsletter to the parents? Not delivered – in week 5 – in week 6 – in week 7 – in week 8”* 1 = yes (in weeks 5–8) 0 = not delivered *“When did you deliver the 1st physical activity behaviour tip-card to the parents? Not delivered – in week 5 – in week 6 – in week 7 – in week 8”* 1 = yes (in weeks 5–8) 0 = not delivered *“When did you deliver the physical activity behaviour poster to the parents? Not delivered – in week 5 – in week 6 – in week 7 – in week 8”* 1 = yes (in weeks 5–8) 0 = not delivered *“All planned activities were performed. Totally disagree – disagree – neither disagree nor agree – agree – totally agree”* 1 = a score of ≥3.52 0 = a score of <3.52 *“Did you implement the classroom activities as described in the manual for physical activity? Never – rarely – sometimes – often – always”* 1 = score ≥ mean value of 3.60 0 = score < mean value of 3.60 *“Was equipment and space appropriately arranged for physical education lessons? Never – rarely – sometimes – often – always”* 1 = score ≥ mean value of 4.18 0 = score < mean value of 4.18	*“How much time did you devote on physical education lessons on an average weekly basis for this month?”* 1 = score ≥ mean value of 122.4 min/week 0 = score < mean value of 122.4 min/week *“Did you devote on average at least one hour per week in the classroom activities as described in the manual? Never – rarely – sometimes – often – always”* 1 = score ≥ mean value of 4.14 0 = score < mean value of 4.14 Sum score of eight items related to classroom activities for physical activity (implementation of 4 kangaroo stories and 4 excursions) (mean score: 1.90) 1 = a score of ≥1.90 0 = a score of <1.90	*“It was easy to read and understand the text in the Classroom Activity Guide for physical activity”* 1 = score ≥ mean value of 4.07 0 = score < mean value of 4.07 *“The amount of information and activities in the Classroom Activity Guide for physical activity were appropriate”* 1 = score ≥ mean value of 3.85 0 = score < mean value of 3.85 *“It was easy to implement the activities described in the Classroom Activity Guide for physical activity”* 1 = score ≥ mean value of 3.67 0 = score < mean value of 3.67 *“I enjoyed the activities I delivered this month”* 1 = score ≥ mean value of 4.09 0 = score < mean value of 4.09 *“The activities I delivered this month were enjoyed by the children”* 1 = score ≥ mean value of 4.28 0 = score < mean value of 4.28 *“The information presented in the Classroom Activity Guide for physical activity, the content of the material and the way the activities should be delivered are appropriate to achieve the goals”* 1 = score ≥ mean value of 4.00 0 = score < mean value of 4.00
Questionnaire Teachers’ physical activity logbook (repetition period)	*“When did you deliver the 2nd physical activity behaviour newsletter to the parents? Not delivered – in week 19 – in week 20”* 1 = yes (in weeks 19–20) 0 = not delivered *“When did you deliver the 2nd physical activity behaviour tip-card to the parents? Not delivered – in week 19 – in week 20”* 1 = yes (in weeks 19–20) 0 = not delivered *“All planned activities were performed. Totally disagree – disagree – neither disagree nor agree – agree – totally agree”* 1 = a score of ≥3.44 0 = a score of <3.44 *“Did you implement the classroom activities as described in the manual for physical activity? Never – rarely – sometimes – often – always”* 1 = score ≥ mean value of 3.58 0 = score < mean value of 3.58 *“Was equipment and space appropriately arranged for physical education lessons? Never – rarely – sometimes – often – always”* 1 = score ≥ mean value of 4.31 0 = score < mean value of 4.31	*“How much time did you devote on physical education lessons on an average weekly basis for this month?”* 1 = score ≥ mean value of 123.3 min/week 0 = score < mean value of 123.3 min/week *“Did you devote on average at least one hour per week in the classroom activities as described in the manual? Never – rarely – sometimes – often – always”* 1 = score ≥ mean value of 3.82 0 = score < mean value of 3.82 Sum score of eight items related to classroom activities for physical activity (implementation of 4 kangaroo stories and 4 excursions) (mean score: 1.55) 1 = a score of ≥1.55 0 = a score of <1.55	*“It was easy to implement the activities described in the Classroom Activity Guide for physical activity”* 1 = score ≥ mean value of 3.68 0 = score < mean value of 3.68 *“I enjoyed the activities I delivered this month”* 1 = score ≥ mean value of 3.92 0 = score < mean value of 3.92 *“The activities I delivered this month were enjoyed by the children”* 1 = score ≥ mean value of 4.12 0 = score < mean value of 4.12
Mean score (± standard deviation)/maximum score	6.23 (±1.13)/11	1.55 (±1.13)/6	5.45 (±2.25)/9

### Process evaluation: parents/caregivers

Also parents/caregivers had to fill in a questionnaire regarding the process of the PA component of the intervention. These questions were also based on the model of Saunders et al. (2005) [25, 26]. The key process evaluation elements that were questioned in the questionnaire for parents were “Dose delivered”, “Dose received – exposure” and “Dose received – satisfaction” [25, 26]. No information on “Fidelity”, “Reach” and “Context” was available.

The specific questions per key element that were used from the questionnaire are depicted in Table 2 with an explanation about the score. The questions with answer possibilities on a 5-point-scale were dichotomised based on the mean score. All questions with a score of 0 or 1 were used to calculate the total process evaluation score for each parent/caregiver by adding up all scores. Again, a higher process evaluation score represents a higher level of the intervention and satisfaction process. The minimum score was 0 and the maximum score was 17. Based on the parents’/caregivers’ process evaluation scores, four- to six-year-old children were divided into three groups, based on tertiles: four- to six-year-old children with a low (recoded into 1), a medium (recoded into 2) or a high level (recoded into 3) of parental process evaluation score. To look for a difference in effect between the intervention group (with three categories: 1, 2, 3) and the control group (recoded into 0), the control group was again added as an extra level of implementation and satisfaction.

**Table 2:** Overview process evaluation questions to calculate the process evaluation score (score on 17) for parents/caregivers

Dose delivered = whether the intervention was delivered as intended by the parents/caregivers and received by the children	Dose received – exposure = whether the intervention was delivered as intended by the teachers and received by the parents/caregivers	Dose received – satisfaction = how the intervention was received by the parents/caregivers
*“Did you implement the suggested activities of the ToyBox Newsletters and Tip-cards? Never – rarely – sometimes – often – always”* 1 = score ≥ mean value of 3.07 0 = score < mean value of 3.07	*“Did you or your partner receive the materials regarding physical activity?”* (one score for each component: Newsletter 1, Newsletter 2, Tip-card 1, Tip-card 2, Poster) 1 = yes 0 = no & I don’t know *“Did you or your partner read the materials regarding physical activity?”* (one score for each component: Newsletter 1, Newsletter 2, Tip-card 1, Tip-card 2, Poster) 1 = yes 0 = no & I don’t know	*“In general, how easy was it to understand the text in the ToyBox Newsletters and Tip Cards? Very difficult – difficult – easy – very easy”* 1 = score ≥ mean value of 3.45 0 = score < mean value of 3.45 *“In general, did you find the information provided in the ToyBox Newsletters and Tip Cards trustful? Not at all – to a little degree – neither trustful or not trustful – to some degree – to a large degree”* 1 = score ≥ mean value of 4.39 0 = score < mean value of 4.39 *“In general, how useful did you find the Suggestions and Tips for parents in the ToyBox Newsletters and Tip Cards? Not useful at all – a little useful – somewhat useful – very useful”* 1 = score ≥ mean value of 3.25 0 = score < mean value of 3.25 *“Did you/your partner and your child enjoy the ToyBox activities conducted with the family? I did not enjoy it at all – I did not enjoy it so much – I enjoyed it – I enjoyed it a lot”* 1 = score ≥ mean value of 2.87 0 = score < mean value of 2.87 *“In general, what did you think about the amount of text in the ToyBox Newsletters and Tip cards? Far too much – too much – about right – too little – far too little”* 1 = 3 0 = > 3 & < 3 *“In general, what did you think of the design (colours, lay out, type of letters) of the ToyBox Newsletters and Tip Cards? I did not like it at all – I did not like it so much – I liked it – I liked it a lot”* 1 = score ≥ mean value of 3.08 0 = score < mean value of 3.08
Mean score (± standard deviation)/maximum score 0.33 (±0.47)/1	Mean score (± standard deviation)/maximum score 4.86 (±4.16)/10	Mean score (± standard deviation)/maximum score 1.88 (±1.50)/6

### Statistical analyses

Steps per day were separately calculated for weekdays, weekend days and average days. To take into account that some four- to six-year-old children had more weekend days than others, outcome variables on an average day were calculated using the following formula: ((MEAN(outcome on weekday 1, outcome on weekday 2)*5) + (MEAN(outcome on weekend day 1, outcome on weekend day 2)*2)/7. Prior to all analyses, all outcome measures were first checked for normal distribution (skewness <0.70) and appeared to be normally distributed. Descriptive statistics were computed to describe the characteristics (age, sex) of the sample, and were reported as frequencies (%) or means and standard deviations or percentages.

Multilevel repeated measures analyses were performed using MLwiN 2.31 (Centre for Multilevel Modelling, University of Bristol, UK) to assess the effectiveness of the intervention on steps per weekday, weekend day and average day. Multilevel modeling (five levels: time, child, kindergarten class, kindergarten, country) was used to take clustering of two measurements (baseline and follow-up) of four- to six-year-old children in kindergarten classes in kindergartens in countries into account. For the country-specific analyses, four levels were used (time, child, kindergarten class, kindergarten). All analyses were adjusted for age and sex. Two ß-values will be reported in the results: (1) the ß-value for ‘time’ is the estimate for the time effect, and can be interpreted as the magnitude of change in the outcome variable going from follow-up to baseline for the reference category (i.e., control group), and (2) the ß-value for ‘time*condition’ is the estimate for the intervention effect for all outcome variables, which describes the difference between the mean change in the intervention group and the mean change in the control group.

To study if the effect was different regarding the intervention process evaluation score four- to six-year old children’s steps per weekday, steps per weekend day and steps per day, multilevel repeated measures analyses (‘time*process evaluation score’) were performed (five levels: time, child, kindergarten class, kindergarten, country). All analyses were adjusted for children’s age and sex. For all analyses, statistical significance level was set at *p* < 0.05.

## Results

### Descriptive results

In total, 2438 children (51.9% boys, mean age at baseline 4.75 ± 0.43 years) provided valid step count data at baseline and follow-up (drop-out of 71.3%). At baseline, four- to six-year-old children took 10,739 (±3258) steps per weekday, 9690 (±3850) steps per weekend day, and 10,440 (±2898) steps on an average day. At follow-up, they took 10,745 (±3501) steps per weekday, 9028 (±3924) steps per weekend day, and 10,254 (±3059) steps on an average day. Descriptive analyses showed that four- to six-year-old children from the control group significantly took less steps at baseline compared to four- to six-year-old children from the control group, both at weekdays (*t* = 8.07, *p* < 0.001) and average days (*t* = 6.78, *p* < 0.001). There was no significant difference for steps at weekend days. In addition, 37.4%, 28.9%, and 32.1% of four- to six-year-old children complied with the PA guidelines of 11,500 steps per day [34] on weekdays, weekend days and average days respectively, at baseline. At follow-up, 38.4%, 24.4% and 31.4% of four- to six-year-old children complied with the PA guidelines on weekdays, weekend days and average days, respectively. The CONSORT checklist can be found in Additional file 1.

### Intervention effects: total sample

Results obtained from the multilevel repeated measures analyses for the PA outcomes in the total sample are shown in Table 3. No significant intervention effects were found for steps per day, steps per weekday and steps per weekend day.

**Table 3:** Time and interaction effects for steps per average day, steps per weekday and steps per weekend day in the total sample (adjusted for age and sex)

*n* = 2438 (*I* = 1605; C = 833)		PRE (steps/day)	POST (steps/day)	Time		Time * condition	
				ß	*p*-value	ß	*p*-value
Average day	I	10,898	10,712				
	C	10,280	10,098	−182.23	0.04	−4.27	0.98
Weekday	I	11,205	11,166				
	C	10,412	10,505	92.83	0.70	−131.91	0.44
Weekend day	I	10,118	9563				
	C	9941	9071	−869.88	<0.001	314.84	0.14

*I* intervention group, *C* control group

Time: the ß-value for ‘time’ is the estimate for the time effect, and can be interpreted as the magnitude of change in the outcome variable going from follow-up to baseline for the reference group (i.e., control group)

Time*Condition: the ß-value for ‘time*condition’ is the estimate for the intervention effect for all outcome variables, which describes the difference between the mean change in the intervention group and the mean change in the control group

### Country-specific intervention effects

Only one significant intervention effect was found for Bulgarian four- to six-year-old children’s steps per day with children from the intervention group having an increase in steps per average day from baseline to follow-up compared to children from the control group who experience a decrease in steps per average day. No other country-specific intervention effects were found, as depicted in Table 4.

**Table 4:** Time and interaction effects for steps per average day, steps per weekday and steps per weekend day for the six European countries (adjusted for age and sex)

		PRE (steps/day)	POST (steps/day)	Time		Time * condition	
				ß	*p*-value	ß	*p*-value
Belgium (*n* = 540; *I* = 332; C = 208)							
Average day	I	10,453	10,705				
	C	10,475	10,455	−20.15	0.08	271.55	0.23
Weekday	I	11,029	11,149				
	C	10,992	10,969	−23.24	0.32	142.76	0.60
Weekend day	I	9028	9493				
	C	9165	9036	−128.64	0.04	593.53	0.11
Bulgaria (*n* = 78; *I* = 33; C = 45)							
Average day	I	7108	7650				
	C	8708	8074	−633.58	0.20	1175.75	0.03
Weekday	I	6677	7231				
	C	8354	7549	−805.00	0.33	1358.65	0.07
Weekend day	I	8471	8986				
	C	9564	9360	−203.52	0.47	718.51	0.44
Germany (*n* = 212; *I* = 180; C = 32)							
Average day	I	11,808	11,766				
	C	12,048	11,130	−917.75	0.88	875.88	0.21
Weekday	I	12,182	12,131				
	C	12,548	11,739	−809.20	0.87	757.60	0.35
Weekend day	I	10,814	10,797				
	C	10,471	9282	−1189.11	0.96	1171.59	0.24
Greece (*n* = 356; *I* = 260; C = 96)							
Average day	I	9943	10,190				
	C	9003	9301	298.22	0.28	−51.38	0.91
Weekday	I	10,394	10,697				
	C	9143	9718	575.37	0.24	−271.78	0.58
Weekend day	I	8822	8927				
	C	8698	8303	−394.65	0.72	499.64	0.38
Poland (*n* = 837; *I* = 512; C = 325)							
Average day	I	11,475	10,969				
	C	10,686	10,615	−70.67	0.003	−434.55	0.11
Weekday	I	11,589	11,526				
	C	10,662	11,065	403.05	0.75	−465.64	0.13
Weekend day	I	11,186	9574				
	C	10,753	9498	−1255.97	<0.001	−356.81	0.36
Spain (*n* = 415; *I* = 288; C = 127)							
Average day	I	12,954	12,269				
	C	11,554	10,803	−751.10	0.001	65.82	0.87
Weekday	I	13,688	13,089				
	C	11,924	11,518	−406.22	0.02	−193.30	0.67
Weekend day	I	11,096	10,197				
	C	10,649	9036	−1613.30	0.003	713.61	0.19

*I* intervention group, *C* control group

Time: the ß-value for ‘time’ is the estimate for the time effect, and can be interpreted as the magnitude of change in the outcome variable going from follow-up to baseline for the reference group (i.e., control group)

Time*Condition: the ß-value for ‘time*condition’ is the estimate for the intervention effect for all outcome variables, which describes the difference between the mean change in the intervention group and the mean change in the control group

### Relationship between the teachers’ process evaluation score and the effects on four- to six-year-old children’s steps per day (total intervention sample)

In total, a teachers’ process evaluation score could be calculated for 1605 children (out of 2438), as not all teachers filled in the monthly logbooks. From the 460 teachers who participated in the intervention, 8.1% (*n* = 41) did not fill in the logbook for PA during the first focus, and 23.3% (*n* = 107) did not fill in the logbook for PA during the repetition period. The mean teachers’ process evaluation score for all intervention kindergartens was 13.49 (±2.56) on a total score of 26 (minimum = 6.50, maximum = 19.00). Kindergartens were divided into three groups, based on the teachers’ process evaluation score: (1) kindergartens with the lowest teachers’ process evaluation score (score 6.50–12.74; n_four- to six-year-old children_ = 501; n_kindergartens_ = 36), (2) kindergartens with a medium teachers’ process evaluation score (score 12.75–14.00; n_four- to six-year-old children_ = 612; n_kindergartens_ = 32), and (3) kindergartens with the highest teachers’ process evaluation score (score 14.01–19.00; n_four- to six-year-old children_ = 492; n_kindergartens_ = 34). Of all 312 teachers, 37.1% had a low, 29.7% had a medium and 33.2% had a high teachers’ process evaluation score. Teachers scored best on dose received (satisfaction; 5.45/9) and fidelity (6.23/11) and had the lowest scores for dose delivered (1.55/6). Detailed information on the scores for each component of the teachers’ process evaluation score can be found in Table 1.

Regarding steps per weekday, a significant interaction effect was found between children from the control group and children with a low teachers’ process evaluation score, going from baseline to follow-up (β = −553.95 (SE = 235.50); *p* = 0.02). Four- to six-year-old children with a low teachers’ process evaluation score significantly decreased their steps per weekday from baseline to follow-up (−461 steps/weekday; *p* = 0.01), while children from the control group did not significantly change their steps per weekday from baseline to follow-up (*p* = 0.52). Furthermore, a significant interaction effect was found between children with a low teachers’ process evaluation score and a medium and high teachers’ process evaluation score, going from baseline to follow-up (β = 558.57 (SE = 250.95), *p* = 0.03 and β = 682.16 (SE = 264.62), *p* = 0.01). Four- to six-year-old children with a medium teachers’ process evaluation score and a high teachers’ process evaluation score did not significantly decrease their steps per weekday going from baseline to follow-up (*p* = 0.56 and *p* = 0.24, respectively), while children with a low teachers’ process evaluation score significantly decreased their steps per weekday (−461 steps/weekday; *p* = 0.01).

Regarding steps per weekend day, a significant interaction effect was found between children from the control group and children with a high teachers’ process evaluation score, going from baseline to follow-up (β = 866.35 (SE = 293.65), *p* = 0.003). Four- to six-year-old children from the control group significantly decreased their steps per weekend day from baseline to follow-up (−870 steps/weekday, *p* < 0.001), while children with a high teachers’ process evaluation score did not (*p* = 0.99). Furthermore, a significant interaction effect was found between children with a low and children with a medium teachers’ process evaluation score (β = −669.56 (SE = 310.93), *p* = 0.03), and between children with a medium and children with a high teachers’ process evaluation score (β = 1095.61 (SE = 312.67), *p* < 0.001). Four- to six-year-old children with a medium teachers’ process evaluation score significantly decreased their steps per weekend day from baseline to follow-up (−1099 steps/weekend day, *p* < 0.001), while children with a low teachers’ process evaluation score and children with a high teachers’ process evaluation score did not show a significant change in their steps between the two time points (*p* = 0.06 and *p* = 0.99, respectively).

**Fig. 3:**
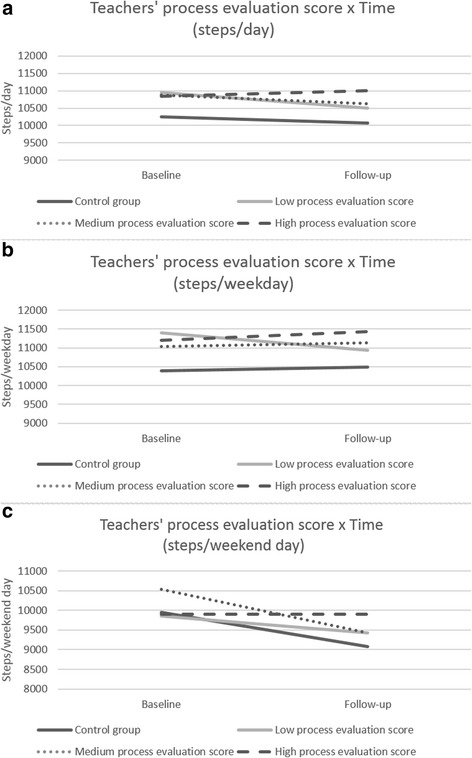
Teachers’ process evaluation score x Time for **a** steps/average day, **b** steps/weekday and **c** steps/weekend day

Regarding steps per day, a significant interaction effect was found between four- to six-year-old children with a low teachers’ process evaluation score and a high teachers’ process evaluation score, going from baseline to follow-up (β = 608.98 (SE = 234.10); *p* = 0.01). This means that children with a low teachers’ process evaluation score significantly decreased their steps per day from baseline to follow-up (−452 steps/day; *p* = 0.01), while children with a high teachers’ process evaluation score did not change their steps per day from baseline to follow-up. No other interaction effects were found for steps per day. All interaction effects between time and teachers’ process evaluation score for steps per weekday, steps per weekend day and steps per day can be found in Fig. 3. In addition, Fig. 3 clearly shows that at baseline, four- to six-year-old children from the control group had a lower amount of steps per weekday and steps per average day compared to the intervention group.

### Relationship between the parents’/caregivers’ process evaluation score and the effects on four- to six-year-old children’ steps per day (total intervention sample)

In total, a parents’/caregivers’ process evaluation score could be calculated for 1420 children (drop-out of 11.5%). The mean parents’/caregivers’ process evaluation score for all intervention kindergartens was 6.90 (±3.50) on a total score of 17 (minimum = 0; maximum = 14). Four- to six-year-old children were divided into three groups, based on the parents’/caregivers’ process evaluation score: (1) four- to six-year-old children with a low parents’/caregivers’ process evaluation score (score 0.00–5.00; n_four- to six-year-old children_ = 464), (2) four- to six-year-old children with a medium parents’/caregivers’ process evaluation score (score 6.00–9.00; n_four- to six-year-old children_ = 498), and (3) four- to six-year-old children with a high parents’/caregivers’ process evaluation score (score 10.00–14.00; n_four- to six-year-old children_ = 458). Of all parents, 31.8% had a low, 34.7% had a medium, and 33.5% had a high parents’/caregivers’ process evaluation score. Parents/caregivers scored best on dose received - exposure (4.86/10) and had the lowest scores for dose received - satisfaction (1.88/6). Detailed information on the scores for each component of the parents’/caregivers’ process evaluation score can be found in Table 2.

For steps per weekday, steps per weekend day and steps per day, no significant interaction effects were found between time and parents’/caregivers’ process evaluation score (all *p* > 0.05). This means that there is no difference between baseline and follow-up in steps per weekday, steps per weekend day or steps per day for four- to six-year-old children from the control group and four- to six-year-old children with a low, medium and high parents’/caregivers’ process evaluation score.

## Discussion

The aim of the present study was twofold. First, we wanted to investigate the effect of the PA-component of the ToyBox-intervention on European four- to six-year-old children’s objectively measured steps per day for the total sample, and for the different countries (Belgium, Bulgaria, Germany, Greece, Poland, and Spain). Second, we wanted to examine whether a higher teachers’ or parents’/caregivers’ process evaluation score was related to more beneficial effects on four- to six-year-old children’s steps per day.

For the total sample, no intervention effects were found, which means that the intervention did not cause an effect on four- to six-year-old children’s PA in terms of steps per day. Also country-specific analyses revealed no effects on European four- to six-year-old children’s steps per day. The lack of effects on children’s PA is in line with other published studies targeting four- to six-year-old children’s PA levels [16, 17]. One might have expected to find larger effects in four- to six-year-old children from Bulgaria and Greece, as these are the countries with the lowest levels of steps per day at baseline [6], and thus have more room to improve their steps per day. However, after analysing the data, it became clear that even in the countries with low steps per day at baseline, the intervention lacked to show an effect. This lack of effect might be due to the age-related decline of PA in children [35]. Furthermore, the ToyBox-intervention focussed on four different behaviours (i.e., water consumption, healthy snacking, sedentary behaviour and PA) during one school year, which means that only a limited period of time (i.e. 6 weeks) was available to implement the PA-module. However, teachers were encouraged to continue the implementation of different parts of the PA-component of the intervention throughout the school year. A recent systematic review and meta-analysis of randomised control trials showed that implementing interventions for a longer period of time (more than 6 months) enhanced intervention effects [36]. It might be suggested for future interventions to focus on PA for a longer period of time to enhance intervention effects. In addition, parents were only passively involved which might also explain the lack of intervention effects, as four- to six-year-old children spend a considerable amount of time in the presence of their parents [21, 27].

The results from the process evaluation showed a medium teachers’ process evaluation score with a mean score of 13.5 on a total of 26. The higher score on fidelity can be explained by the fact that almost all kindergarten teachers handed out the newsletters and tip-cards for the parents/caregivers, based on the set time plan of the ToyBox-intervention. In addition, the higher score for dose received (satisfaction) shows us that kindergarten teachers in general might have enjoyed the intervention components and that they might be convinced about the clarity, appropriateness and the feasibility of the intervention components. The low score for dose delivered can be explained by the fact that the classroom activities were not implemented as intended. Although kindergarten teachers read the Kangaroo stories during the first weeks of intervention implementation, most of them did not implement the proposed excursions. In addition, during the repetition period, less than 25% of kindergarten teachers repeated using the Kangaroo stories and more than 90% of all teachers did not execute the excursions. This shows that excursions might be hard to implement in kindergarten classes, and that the Kangaroo stories can be used for a short period during the intervention, but not for longer implementation periods.

The results in this paper show that there are some differences in effect between the control group and between kindergartens with different process evaluation scores (low to high). For example for steps per weekday, four- to six-year-old children with a medium and high process evaluation score did not decrease their steps from baseline to follow-up, while four- to six-year-old children with a low process evaluation score did. This shows that having teachers that do not implement the intervention as intended (i.e., low teacher process evaluation score) and were not satisfied with the intervention, could have unfavourable effects compared to teachers that implement the intervention in a better way and were satisfied with the intervention or even compared to teachers that did not implement the intervention at all (i.e., control group). This shows that not implementing an intervention (i.e., doing nothing) is better than implementing an intervention with low fidelity to the study protocol. The influence of teachers implementing the intervention as intended (i.e., high teachers’ process evaluation score) was still present on weekend days, as children from the control group decreased their steps per weekend day from baseline to follow-up, while children with a high teachers’ process evaluation score did not. This shows that teachers implementing the intervention with high fidelity to the study protocol can stop a possible decrease in steps per weekday or weekend day. These age-related declines in PA have been studied before [35] and could thus be ameliorated with the ToyBox-intervention, if implemented with high fidelity and well-liked by the teachers. The results from the current study are very relevant, as our results emphasise the major importance of linking process to effect evaluation. These results are comparable to what has already been found in a recent study by Verloigne et al. (2015) in Belgian children (mean age: 6.0 years old) in which favourable effects were found for primary schools with medium and high process evaluation scores of the IDEFICS-intervention, but these effects were not found in preschool children [37]. Other studies found similar results with favourable effects in the targeted behaviours in adolescents with a higher level of intervention implementation [38–40]. However, in the current study, the variation between the three levels of intervention implementation and satisfaction was very small, and the scores were predominantly medium, which might be the cause of the lack of more and stronger effects. Moreover, the context of the kindergarten setting is different compared to the primary school setting, which might explain the differences observed between the studies. There are some possible reasons why the teachers’ process evaluation scores were low in general. In the development of the ToyBox-intervention, focus groups with parents and teachers of four- to six-year-old children were conducted at the beginning of the development-phase, to involve both groups in the development of the intervention [30], which means that they reflected about what could be feasible within an intervention. However, the teachers that were involved in the focus group discussions were not the teachers that eventually had to implement the intervention, which means that focus groups were not used to involve the teachers more in the implementation of the intervention. Furthermore, it might be possible that principals of the intervention kindergartens agreed in participating in the intervention, but that teachers from those kindergartens were not really motivated to implement the intervention (e.g., due to large time investments, busy schedules, etc.). For future interventions, it might be an option to use a participatory health research approach, which means that the target group and the implementers are actively involved in designing and developing an intervention [41, 42]. An option might be to create an interaction with a panel of kindergarten teachers during the development and implementation of the intervention, which enables receiving feedback from the teachers and the development of an intervention that complies with teachers’ skills and takes their needs into consideration. This might lead to better process evaluation scores and thus to better effects. In the ToyBox-intervention, only small flexibility and local adaptations were allowed to ensure comparability of results and in an extend that affected the fidelity of the implementation of certain components of the programme. The implementation of future projects in this field should allow more flexibility to meet the local needs, which is what the study of Hawe et al. (2004) suggests. Hawe et al. (2004) recommends to adapt the intervention to the local context. Rather than standardising the components of the intervention, these components could be seen as “mechanisms” in the change process, and can thus take on different forms according to the local context [43].

A low parents’/caregivers’ process evaluation score was found with a mean score of 6.90 on a total score of 17. The results suggest that parents/caregivers read and were satisfied with the newsletters, tip-cards and posters for the PA component of the ToyBox-intervention, but that they were not satisfied with for example the usefulness, design and the amount of text of these tools. In addition, results showed that there was no difference in effect between four- to six-year-old children from the control group and four- to six-year-old children with a low, medium or high parents’/caregivers’ process evaluation score. This is comparable with the IDEFICS-intervention in which higher levels of parental exposure to the intervention was not related to more favourable effects in children’s BMI z-scores, and parental exposure and involvement in the intervention was much less than aimed for [44]. In the ToyBox-intervention, parents/caregivers were only passively involved as they only received newsletters, tip-cards and posters which they had to read. It might thus be possible that passively involving parents/caregivers in increasing four- to six-year-old children’s steps per day might not be the way to go forward. Actively involving parents/caregivers might be a more promising strategy in future interventions targeting four- to six-year-old children’s steps per day [45–47], and might induce an increase in children’s PA.

Based on the information received from both kindergarten teachers and parents/caregivers, the ToyBox-material and/or components could be adapted and revised to meet the comments. For example by providing active toys and/or activity videos for the teachers. In addition, qualitative research could be helpful to investigate kindergarten teachers’ and parents’/caregivers’ perspectives on what worked and what did not work. The revised material and/or components could then be pilot tested to see whether teachers and parents/caregivers are more satisfied with the intervention components and implement the intervention as intended.

Study strengths are the large sample of four- to six-year-old children that provided valid pedometer data, and the cluster randomised controlled trial with a pre-test post-test design. However, future studies could look into objectively measuring four- to six-year-olds’ PA by using hip and wrist accelerometers simultaneously as wrist-worn accelerometers have shown to measure different PA intensities more accurately [48]. Another strength is the use of process evaluation questionnaires for both kindergarten teachers and parents/caregivers. Key elements from the process evaluation model of Saunders et al. (2005) were used to calculate the process evaluation scores for teachers and parents/caregivers, which shows that process evaluation scores were theory-based [25, 26].

Study limitations include the fact that pedometers cannot distinguish between the different PA intensities and are only able to measure in steps per day. Missing data (e.g., during water-based activities) were not imputed, which may have induced bias. Furthermore, there was a large drop-out of four- to six-year-old children due to a lack of valid pedometer data at both baseline and follow-up. Also, there was no objective measurement of intervention implementation as the data for the process evaluation scores were self-reported in both teachers and parents/caregivers. It is possible that both teachers and parents/caregivers overestimated their efforts, which might have introduced bias. In addition, as not all teachers filled in all logbooks and not all parents filled in the process evaluation questionnaire, this also might have induced bias. Future studies could try to incorporate objective methods (e.g., observation) to study process evaluation. Furthermore, teachers’ logbooks did not contain specific questions regarding the implementation of the environmental changes in the classroom, which means that valuable information was not recorded. This could however be taken into account by using observations. Another limitation that should be considered is that the differences by countries regarding the process evaluation score and sub-scores of each process evaluation component were not reported. However, this manuscript already gives a general image of the link between effect and process evaluation for all countries combined. Finally, it should be acknowledged that there is no standardised way to calculate process evaluation scores. Although it was based on a method used in previous studies [37, 49, 50], there are still some limitations linked to the method used to calculate the process evaluation scores as not all components of Saunders et al. (2005) were used to calculate the score, and equal weights for each component were used. In addition, there is not yet a consensus on how to operationalise the different process evaluation components [37, 51]. Consequently, the process evaluation scores provide a more general idea of the level of process evaluation of the kindergarten teachers and parents/caregivers. Process evaluation has many components, but implementation and satisfaction were the two that were chosen as the focus for this study. In addition, the current study chose to include satisfaction in the process evaluation score, while other papers in the literature have only included implementation scores to study the relationship between process and effect evaluation.

## Conclusion

Overall, the ToyBox-intervention had no effect on European four- to six-year-old children’s steps per day. Future European interventions with the aim to increase four- to six-year-old children’s steps per day should incorporate country-specific adaptations to increase the effectiveness. The process evaluation scores showed that higher teachers’ process evaluation score did not cause a decrease in steps per day, while four- to six-year-old children with a lower score or from the control group did. Therefore, future interventions should search for strategies to motivate the kindergarten teachers to induce larger effects, for instance by using a participatory approach in which kindergarten teachers are involved from the beginning of the study or by providing more active toys and exercise videos. However, this should be combined with actively involving children’s parents.

## Additional file

Additional file 1: CONSORT checklist. (DOCX 23 kb)

## Abbreviations

CONSORT: Consolidated standards of reporting trials
PA: Physical activity
SES: Socio-economic status
