# Supplementary material for: Effect and process evaluation of a kindergarten-based, family-involved cluster randomised controlled trial in six European countries on four- to six-year-old children’s steps per day: the ToyBox-study
Source: Int J Behav Nutr Phys Act. 2017 Aug 29;14:116. doi: 10.1186/s12966-017-0574-z (PMC5576230; doi:10.1186/s12966-017-0574-z)
Supplement: Additional file 1: — CONSORT checklist. (DOCX 23 kb) [file 12966_2017_574_MOESM1_ESM.docx]

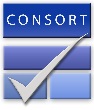
CONSORT 2010 checklist of information to include when reporting a randomised trial*

| Section/Topic | Item No | Checklist item | Reported on page No |
| --- | --- | --- | --- |
| Title and abstract | | | |
|  | 1a | Identification as a randomised trial in the title | Lines 1-2 |
|  | 1b | Structured summary of trial design, methods, results, and conclusions (for specific guidance see CONSORT for abstracts) | Lines 27-49 |
| Introduction | | | |
| Background and objectives | 2a | Scientific background and explanation of rationale | Lines 51-82 |
|  | 2b | Specific objectives or hypotheses | Lines 83-87 |
| Methods | | | |
| Trial design | 3a | Description of trial design (such as parallel, factorial) including allocation ratio | Lines 92-95; Line 116-118 |
|  | 3b | Important changes to methods after trial commencement (such as eligibility criteria), with reasons | Not applicable |
| Participants | 4a | Eligibility criteria for participants | Lines 101-116 |
|  | 4b | Settings and locations where the data were collected | Lines 125-131 |
| Interventions | 5 | The interventions for each group with sufficient details to allow replication, including how and when they were actually administered | Lines 147-172 |
| Outcomes | 6a | Completely defined pre-specified primary and secondary outcome measures, including how and when they were assessed | Lines 175-181; 184-194; 197-215; 219-233; 236-260 |
|  | 6b | Any changes to trial outcomes after the trial commenced, with reasons | Not applicable |
| Sample size | 7a | How sample size was determined | Lines 115-116 |
|  | 7b | When applicable, explanation of any interim analyses and stopping guidelines | Not applicable |
| Randomisation: |  |  |  |
| Sequence generation | 8a | Method used to generate the random allocation sequence | Lines 117-121 |
|  | 8b | Type of randomisation; details of any restriction (such as blocking and block size) | Lines 117-121 |
| Allocation concealment mechanism | 9 | Mechanism used to implement the random allocation sequence (such as sequentially numbered containers), describing any steps taken to conceal the sequence until interventions were assigned | Lines 119-121 |
| Implementation | 10 | Who generated the random allocation sequence, who enrolled participants, and who assigned participants to interventions | Lines 119-121 |
| Blinding | 11a | If done, who was blinded after assignment to interventions (for example, participants, care providers, those assessing outcomes) and how | Not applicable |
|  | 11b | If relevant, description of the similarity of interventions | Not applicable |
| Statistical methods | 12a | Statistical methods used to compare groups for primary and secondary outcomes | Lines 236-260 |
|  | 12b | Methods for additional analyses, such as subgroup analyses and adjusted analyses | Lines 236-260 |
| Results | | | |
| Participant flow (a diagram is strongly recommended) | 13a | For each group, the numbers of participants who were randomly assigned, received intended treatment, and were analysed for the primary outcome | Lines 264-269 & Figure 1 |
|  | 13b | For each group, losses and exclusions after randomisation, together with reasons | Figure 1 |
| Recruitment | 14a | Dates defining the periods of recruitment and follow-up | Lines 125-131 |
|  | 14b | Why the trial ended or was stopped | Not applicable |
| Baseline data | 15 | A table showing baseline demographic and clinical characteristics for each group | Not applicable |
| Numbers analysed | 16 | For each group, number of participants (denominator) included in each analysis and whether the analysis was by original assigned groups | Tables 1-2 |
| Outcomes and estimation | 17a | For each primary and secondary outcome, results for each group, and the estimated effect size and its precision (such as 95% confidence interval) | Tables 1-2 |
|  | 17b | For binary outcomes, presentation of both absolute and relative effect sizes is recommended | Not applicable |
| Ancillary analyses | 18 | Results of any other analyses performed, including subgroup analyses and adjusted analyses, distinguishing pre-specified from exploratory | Figure 2a, 2b, 2c |
| Harms | 19 | All important harms or unintended effects in each group (for specific guidance see CONSORT for harms) | Not applicable |
| Discussion | | | |
| Limitations | 20 | Trial limitations, addressing sources of potential bias, imprecision, and, if relevant, multiplicity of analyses | Lines 485-496 |
| Generalisability | 21 | Generalisability (external validity, applicability) of the trial findings | Lines 371-479 |
| Interpretation | 22 | Interpretation consistent with results, balancing benefits and harms, and considering other relevant evidence | Lines 371-479 |
| Other information | | |  |
| Registration | 23 | Registration number and name of trial registry | Lines 139-140 |
| Protocol | 24 | Where the full trial protocol can be accessed, if available | [www.toybox-study.eu](http://www.toybox-study.eu) |
| Funding | 25 | Sources of funding and other support (such as supply of drugs), role of funders | Line 508-511 |

*We strongly recommend reading this statement in conjunction with the CONSORT 2010 Explanation and Elaboration for important clarifications on all the items. If relevant, we also recommend reading CONSORT extensions for cluster randomised trials, non-inferiority and equivalence trials, non-pharmacological treatments, herbal interventions, and pragmatic trials. Additional extensions are forthcoming: for those and for up to date references relevant to this checklist, see [www.consort-statement.org](http://www.consort-statement.org).
